# Incidence of tuberculosis in HIV-infected adults on first- and second-line antiretroviral therapy in India

**DOI:** 10.1186/s12879-019-4569-z

**Published:** 2019-10-29

**Authors:** Akshay N. Gupte, Dileep Kadam, Shashikala Sangle, Bharat B. Rewari, Sonali Salvi, Amol Chavan, Smita Nimkar, Jonathan Golub, Nikhil Gupte, Amita Gupta, Ivan Marbaniang, Vidya Mave

**Affiliations:** 1 Johns Hopkins University School of Medicine, 600 N Wolfe Street, Phipps 521, Baltimore, MD 21287 USA; 2 Byramjee-Jeejeebhoy Medical College, Pune, India; 3 Former National Programme Office, National AIDS Control Organization, New Delhi, India; 4 Byramjee-Jeejeebhoy Medical College Clinical Trials Unit, Pune, India

**Keywords:** HIV, Tuberculosis, Second-line ART, Competing risks, India

## Abstract

**Background:**

Programmatic data on the baseline risk of tuberculosis in people living with HIV (PLHIV) are needed to evaluate long-term effectiveness of the ongoing isoniazid preventive therapy (IPT) roll-out in India.

**Methods:**

We estimated the incidence rate and risk factors of tuberculosis disease in adult PLHIV initiating first- and second-line anti-retroviral therapy (ART) prior to widespread IPT in a public ART center in Pune, India.

**Results:**

4067 participants contributing 5205.7 person-years of follow-up on first-line ART and 871 participants contributing 1031.7 person-years of follow-up on second-line ART were included in the analysis. The incidence rate of tuberculosis was 4.39 cases (95%CI 3.86–5.00) per 100 person-years on first-line ART and 1.64 cases (95%CI 1.01–2.63) per 100 person-years on second-line ART (*p* < 0.001). After adjusting for competing risks, male sex (aSHR = 1.33, 95%CI 1.02–1.74, *p* = 0.03), urban residence (aSHR = 1.53, 95%CI 1.13–2.07, *p* = 0.006) and CD4+ counts < 350 cells/mm^3^ (aSHR = 3.06 vs CD4 > 350 cells/mm^3^, 95%CI 1.58–5.94, p < 0.001) at ART initiation were associated with higher risk of tuberculosis independent of ART regimen.

**Conclusion:**

Risk of tuberculosis was lower in PLHIV receiving second-line ART compared to first-line ART. Prioritizing IPT in PLHIV with low CD4+ counts, urban residence and in males may further mitigate the risk of tuberculosis during ART.

## Background

Tuberculosis disease (TB) has surpassed HIV as the leading infectious cause of mortality globally. An estimated one-third of the world’s population is infected with *Mycobacterium tuberculosis*, over 10 million developed TB and 1.5 million died in 2017. People living with HIV (PLHIV) are at high risk of developing TB and account for nearly 9% of new TB cases and nearly 300,000 TB-related deaths globally [1].

Widespread anti-retroviral therapy (ART) has significantly reduced the incidence of TB in PLHIV. However, the burden of TB remains high in this population with prevalence ranging from 3 to 72% in high burden settings [2]. While first-line ART regimens containing a non-nucleotide reverse transcriptase inhibitor (NNRTI) have significantly reduced the burden of AIDS and non-AIDS defining events, treatment failure can occur in nearly 20% of patients, requiring a switch to a second-line protease inhibitor (PI)-based regimen [3]. PLHIV receiving second-line regimens represent a growing and potentially high-risk population for TB.

To further reduce the burden of TB, the World Health Organization (WHO) recommends isoniazid prevention therapy (IPT) for all PLHIV in high TB-burden settings [4]. India has the third highest HIV burden and the highest TB burden globally with nearly one million new TB cases among PLHIV each year [5]. India’s National AIDS Control Organization (NACO) provides free first- and second-line ART, and has recommended IPT in all PLHIV since December 2016 [6]. However, programmatic data on the risk of TB in PLHIV receiving first- and second-line ART in India are limited. Documenting the burden and risk factors of TB in this population, especially prior to widespread IPT implementation, will provide a baseline estimate to compare the long-term effectiveness of IPT and, inform prioritized phasing in of IPT to reduce the burden of TB among PLHIV in India.

Therefore, we conducted a retrospective cohort study using programmatic data from one of India’s largest public sector ART delivery programs in Pune, Maharashtra. Our study aimed to estimate the incidence rate and risk factors of TB among HIV-infected adults receiving first- and second-line ART in the absence of widespread IPT.

## Methods

### Study design and setting

We conducted a retrospective cohort study of HIV-infected adults registered at the Byramjee-Jeejeebhoy Government Medical College-Sassoon General Hospitals (BJGMC-SGH) ART center in Pune, India. BJGMC-SGH is a public tertiary referral hospital and one of the largest NACO supported ART centers in India serving the population of Pune city and its surrounding semi-urban and rural region, with over 37,000 registered HIV-infected patients and nearly 1200 patients on second-line PI-based ART regimens. All patients registered at the BJGMC-SGH ART center received free clinical care and treatment as per national program guidelines [7]. Prior to June 2016, ART was initiated in HIV-infected adults with a CD4+ cell count below 350 cells/mm^3^ regardless of symptoms, or in the event of an opportunistic infection, including TB, regardless of CD4+ cell counts. These guidelines changed in June 2016 and ART was initiated in HIV-infected adults with a CD4+ cell count below 500 cells/mm^3^, or in the event of an opportunistic infection regardless of CD4+ cell counts thereafter. During the study period, standard first-line ART included lamivudine, zidovudine or stavudine, and nevirapine or efavirenz based regimens. Tenofavir was included on a case-by-case basis. All patients underwent monthly clinical evaluations. The primary criterion for initiating second-line ART was a viral load (VL) above 5000 copies/mL; however, TB was also considered a criterion at the discretion of the treating physician. Standard second-line ART included a boosted atazanavir-based PI regimen, except in the case of known toxicity or adverse events where a boosted lopinavir-based PI regimen was prescribed. Nucleoside reverse transcriptase inhibitors were selected on a case-by-case basis and depended on the patient’s first-line regimen. National guidelines recommending IPT for all PLHIV were implemented at the BJGMC-SGH ART center starting April 2017.

### Study procedures

Data for this analysis were extracted from existing BJGMC-SGH ART center databases. We identified adults (≥18 years) who initiated first- or second-line ART from January 2010 to December 2016 at the BJGMC-SGH ART center for inclusion in our study. We excluded participants with prevalent TB, defined as a clinical (symptom and/or chest radiograph evaluation suggestive of TB) or microbiological (Acid Fast Bacilli [AFB] on smear microscopy) diagnosis of TB or receiving TB therapy at first- or second-line ART initiation. We additionally excluded participants who were transferred to another ART center for treatment within one day of ART initiation as these participants would not be available for follow-up at the BJGMC-SGH ART clinic. Socio-demographic and clinical characteristics, and CD4+ cell counts closest to first- or second-line ART initiation were extracted for analysis. Participants with unavailable data at ART initiation were excluded. Urban residence was defined as having a residential address within the Pune city zonal limits.

All participants at the ART center underwent screening for TB at each clinic visit using a WHO recommended questionnaire to assess symptoms of current cough, night sweats, weight loss and fever. Participants testing positive on the symptom screening questionnaire underwent chest radiography and AFB smear microscopy on at least two clinical samples for confirmation of suspected TB. The final diagnosis of TB recorded in the ART center database was extracted for analysis. Participants lost to follow-up, defined according to NACO guidelines as missing three consecutive monthly clinic visits, were identified from the ART center database. Similarly, data on all-cause mortality was extracted from the ART center databases.

The Institutional Review Boards of Johns Hopkins University and BJGMC-SGH approved the project.

### Statistical analysis

The primary outcome of our study was incident TB disease in participants on first- or second-line ART. The date of TB treatment initiation entered in the participant medical record was used as a proxy for the date of TB diagnosis. Person-time at risk on first- or second-line ART was calculated from the date of respective ART initiation until the occurrence of the first mutually exclusive event of incident TB, death, transfer to another ART center, loss to follow-up, administrative censoring on 31st December 2016 or date of second-line ART initiation among participants who failed first-line ART. Participants initially receiving first-line ART who subsequently received second-line ART did not contribute person-time for second-line ART analysis. Participants who developed TB and died were assigned an outcome of incident TB. Incidence rate (IR) was calculated as the number of incident TB cases divided by the person-time at risk and expressed as events per 100 person-years with accompanying Poisson exact 95% confidence intervals (CI). Kaplan-Meier survival analysis was used to estimate the proportion of participants on first- and second-line ART who remained free of TB after stratifying by CD4+ cell counts at initiation of first- and second-line ART respectively. We identified risk factors for incident TB in participants on first- and second-line ART using all-cause death and loss to follow-up (LTFU) as competing risks to address a potential bias induced by differential outcome ascertainment commonly seen in programmatic data. Sub-distribution hazard ratios (SHR) were estimated using univariable and multivariable competing risks regression previously described by Fine and Gray [8]. Multivariable regression models included baseline participant characteristics such as age, sex, CD4+ cell counts at respective ART initiation and urban residence. Additionally, we included duration on first-line ART prior to second-line ART initiation. Categorical data were summarized as proportions and compared using Fisher’s exact test. Continuous data were summarized as medians with accompanying interquartile range (IQR) and compared using the Wilcoxon rank-sum or Kruskal-Wallis test. *P*-values less than 0.05 were considered statistically significant. Data were analyzed in Stata 15 (StataCorp, Texas).

## Results

**Fig. 1:**
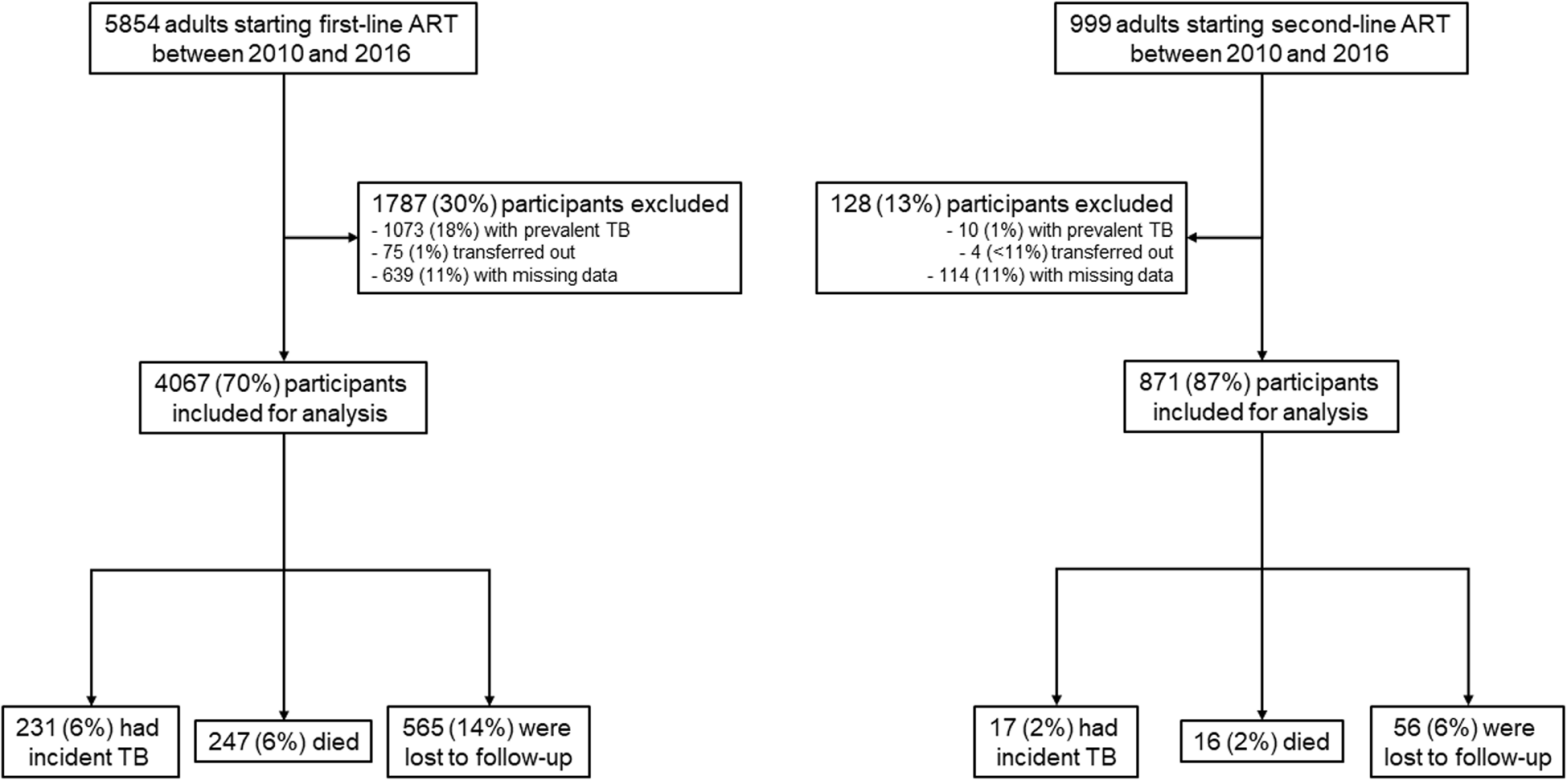
Consort diagram of participant enrollment. ART – antiretroviral therapy, TB – tuberculosis

We identified 5854 and 999 adults initiating first- and second-line ART between January 2010 and December 2016, respectively (Fig. 1). We excluded 1073 (18%) and 10 (1%) participants on first- and second-line ART with prevalent TB, respectively. An additional 79 (2%) participants who were transferred to another ART center and 753 (13%) participants with unavailable socio-demographic or CD4+ data at their respective ART initiation were excluded. Overall, 4067 participants contributing 5205.7 person-years of follow-up on first-line ART and 871 participants contributing 1031.7 person-years of follow-up on second-line ART were included in the analysis. The median (IQR) follow-up time was 12 (4–25) and 15 (11–18) months on first- and second-line ART, respectively (Table 1).

**Table 1:** Participant characteristics by first- and second-line ART receipt

Characteristics	Overall (*n* = 4938)	First-line ART (*n* = 4067)	Second-line ART (*n* = 871)	p-value first- vs second-line ART
Age, median (IQR)	36 (30–43)	36 (30–42)	40 (34–45)	< 0.001
Male, n (%)	2587 (52)	2018 (50)	569 (65)	< 0.001
CD4+ count/mm^3^ at respective ART initiation, median (IQR)	201 (107–298)	184 (97–265)	313 (182–506)	< 0.001
Urban residence, n (%)	3408 (69)	2874 (71)	534 (62)	< 0.001
Follow-up duration (months), median (IQR)	13 (5–23)	12 (4–25)	15 (11–18)	< 0.001
Prior TB	53 (1)	27 (1)	26 (3)	< 0.001
Incident TB, n (%)	248 (5)	231 (6)	17 (2)	< 0.001
Died, n (%)	288 (6)	270 (7)	18 (2)	< 0.001
Lost to follow-up, n (%)	621 (13)	565 (14)	56 (6)	< 0.001

*ART* Antiretroviral therapy, *IQR* Interquartile range, n – frequency

Among participants on first-line ART, 231 (6%) developed TB, 247 (6%) died and 565 (14%) were lost to follow-up. Among participants on second-line ART, 17 (2%) developed TB, 16 (2%) died and 56 (6%) were lost to follow-up (Fig. 1). Compared to participants on first-line ART, those receiving second-line ART were older (median [IRQ] age 36 [30–42] vs 40 [34–45] years), more likely to be male (50% vs 65%), less like to live in an urban setting (71% vs 62%), more likely to have had TB in the past (1% vs 3%) and had higher CD4+ cell counts (median [IQR] CD4+ 184 [97–265] vs 313 [182–506] cells/mm^3^) at their respective ART initiation (*p* < 0.001 for all comparisons) (Table 1).

**Fig. 2:**
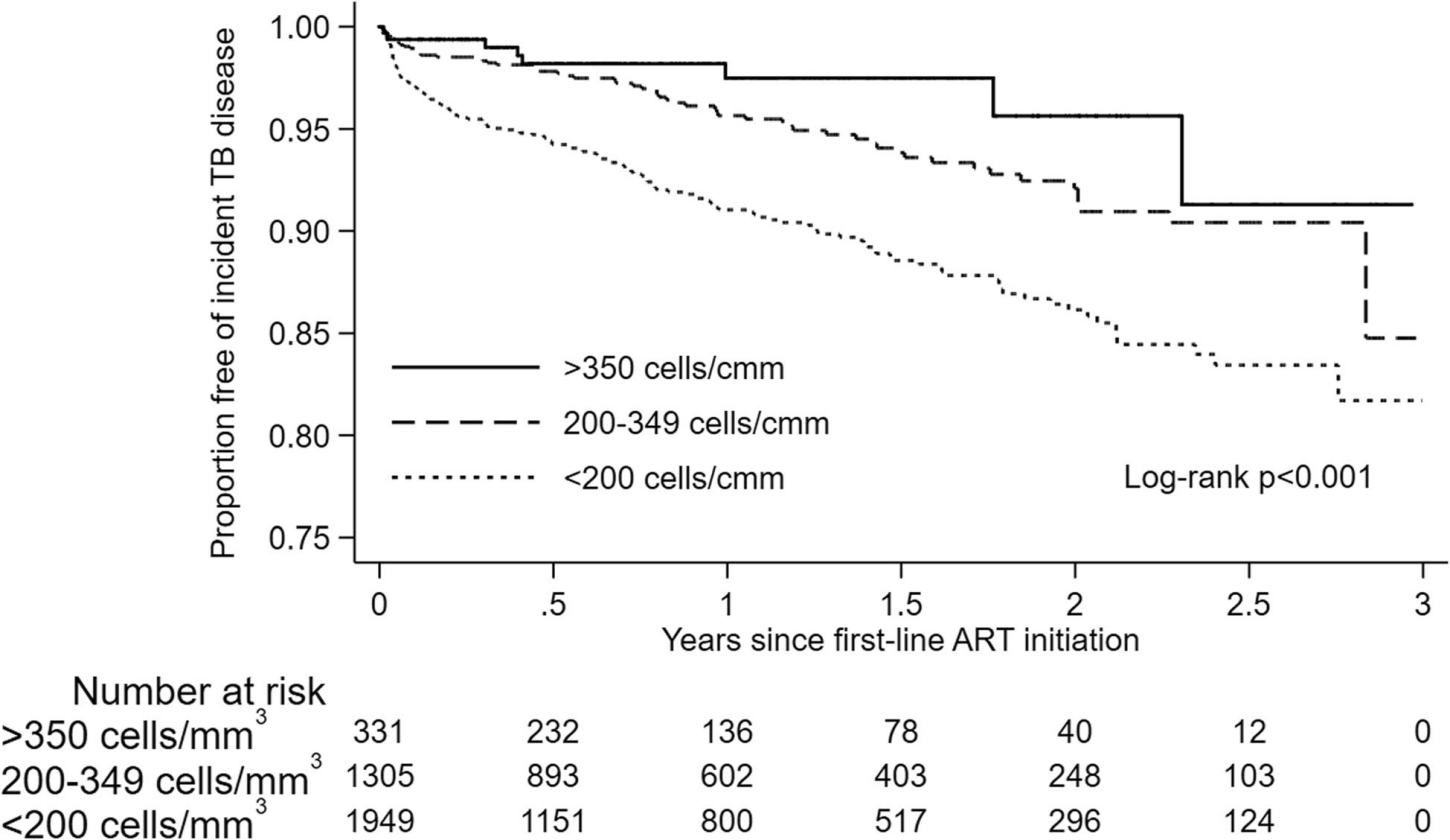
Proportion of participants on first-line ART surviving free of incident TB disease stratified by CD4+ cell counts. Cmm – cubic millimeter, TB – tuberculosis, ART – antiretroviral therapy

**Fig. 3:**
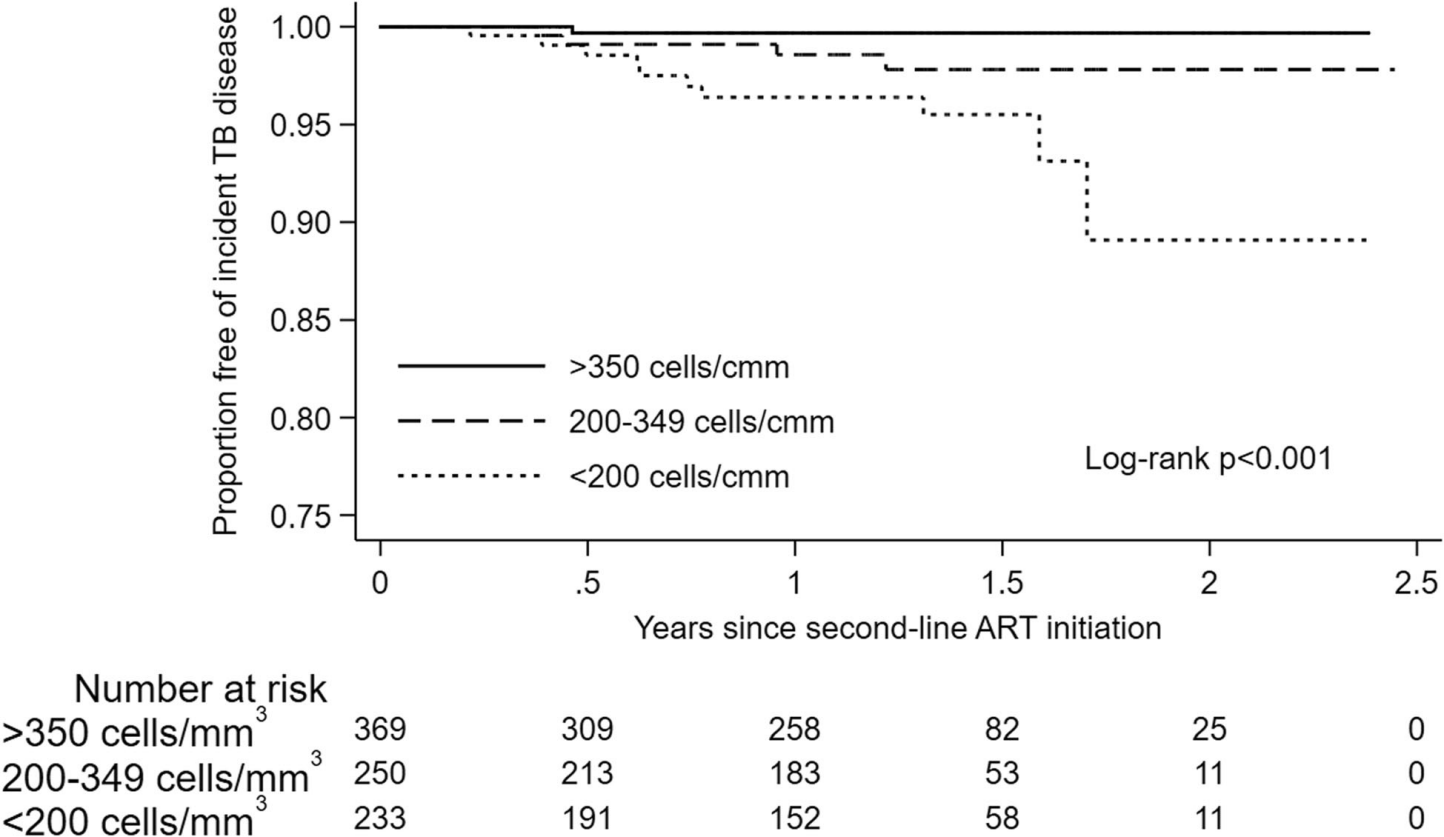
Proportion of participants on second-line ART surviving free of incident TB disease stratified by CD4+ cell counts. Cmm – cubic millimeter, TB – tuberculosis, ART – antiretroviral therapy

The overall incidence rate of TB in our study was 3.94 cases (95%CI 3.48–4.46) per 100 person-years. The incidence rate of TB among participants on first-line ART was 4.39 cases (95%CI 3.86–5.00) per 100 person-years, significantly higher than 1.64 cases (95%CI 1.01–2.63) per 100 person-years among participants on second-line ART (*p* < 0.001). The incidence rate of TB was highest during the first 3 months of first- or second-line ART initiation (Table 2). Participants on second-line ART had lower incidence rates of TB compared to those on first-line ART after stratifying by age, sex, CD4+ cell count at their respective ART initiation, time since their respective ART initiation and type of residence (Table 2). Overall, 119 of 231 (52%) and 5 of 17 (29%) TB cases occurred within 6 months of initiating first- and second-line ART, respectively. Earlier occurrence of TB was more common in participants with CD4+ counts below 350 cells/mm^3^ (p < 0.001) (Figs. 2 and 3).

**Table 2:** Incidence rate of TB disease per 100 person-years in participants receiving first- and second-line ART

Characteristics	Overall		First-line ART		Second-line ART		p-value first- vs second-line ART
	IR	95%CI	IR	95%CI	IR	95%CI	
Overall	3.94	3.48–4.46	4.39	3.86–5.00	1.64	1.01–2.63	< 0.001
Age							
18–29	3.67	2.75–4.88	3.82	2.83–5.16	2.55	0.95–6.79	0.44
30–39	4.10	3.39–4.96	4.39	3.61–5.35	2.25	1.12–4.51	0.07
40–49	3.92	3.11–4.95	4.72	3.71–6.01	1.21	0.50–2.92	0.004
> 50	3.85	2.56–5.80	4.78	3.17–7.19	0	–	–
Sex							
Female	3.26	2.69–3.95	3.46	2.84–4.22	1.67	0.75–3.72	0.08
Male	4.63	3.94–5.46	5.48	4.62–6.49	1.62	0.89–2.92	< 0.001
CD4+ count at respective ART initiation (cells/mm^3^)							
> 350	1.14	0.61–2.12	1.94	0.97–3.89	0.42	0.10–1.71	0.06
S200–349	2.86	2.25–3.64	3.05	2.37–3.91	1.64	0.68–3.96	0.18
< 200	5.55	4.78–6.45	5.72	4.90–6.68	3.74	2.01–6.95	0.18
Time since respective ART initiation							
< 3 months	112.77	92.23–137.89	122.22	99.85–149.60	13.63	1.92–96.82	0.02
3 to 6 months	17.23	11.97–24.79	17.49	11.81–25.88	15.78	5.92–42.04	0.84
> 6 months	2.05	1.72–2.44	2.22	1.84–2.67	1.19	0.67–2.10	0.04
Residence							
Urban	4.31	3.74–4.96	4.63	4.00–5.36	2.37	1.43–3.93	0.01
Semi-urban or rural	3.01	2.31–3.94	3.74	2.85–4.91	0.50	0.12–2.00	0.006

*ART* Antiretroviral therapy, *IR* Incidence rate, *CI* Confidence interval

Multivariable regression analysis with death and loss to follow-up as competing risks identified male sex (aSHR = 1.35, 95%CI 1.03–1.77, *p* = 0.02), CD4+ count less than 200 cells/mm^3^ at ART initiation (aSHR = 3.03 vs CD4+ count > 350 cells/mm^3^, 95%CI 1.48–6.17, *p* = 0.002) and urban residence (aSHR = 1.42 vs non-urban residence, 95%CI 1.04–1.94, p = 0.02) as independent risk factors for TB among participants on first-line ART. Similarly, CD4+ count less than 200 cells/mm^3^ at ART initiation (aSHR = 9.95 vs CD4+ count > 350 cells/mm^3^, 95%CI 2.38–41.60, p = 0.002) and urban residence (aSHR = 4.60 vs non-urban residence, 95%CI 0.96–21.86, *p* = 0.05) were independently associated with higher risk of TB among participants on second-line ART (Table 3).

**Table 3:** Risk factors of incident TB disease in participants receiving first-line ART with death and loss to follow-up as competing risks

Characteristics	Overall				First-line ART				Second-line ART			
	Univariable		Multivariable		Univariable		Multivariable		Univariable		Multivariable	
	SHR (95%CI)	p-value	SHR (95%CI)	p-value	SHR (95%CI)	p-value	SHR (95%CI)	p-value	SHR (95%CI)	p-value	SHR (95%CI)	p-value
Age												
18–29	Ref		Ref		Ref		Ref		Ref		Ref	
30–39	1.09 (0.78–1.54)	0.58	1.00 (0.70–1.41)	0.99	1.15 (0.80–1.64)	0.44	1.02 (0.71–1.47)	0.90	0.92 (0.27–3.08)	0.89	0.95 (0.27–3.34)	0.94
40–49	1.00 (0.69–1.45)	0.96	0.91 (0.62–1.33)	0.63	1.17 (0.80–1.72)	0.40	0.96 (0.65–1.43)	0.87	0.39 (0.10–1.44)	0.15	0.41 (0.08–1.90)	0.25
> 50	0.94 (0.57–1.54)	0.80	0.84 (0.50–1.41)	0.51	1.12 (0.67–1.86)	0.64	0.94 (0.55–1.59)	0.83	–	–	–	–
Sex												
Female	Ref		Ref		Ref		Ref		Ref		Ref	
Male	1.31 (1.02–1.68)	0.03	1.33 (1.02–1.74)	0.03	1.44 (1.11–1.87)	0.006	1.35 (1.03–1.77)	0.02	0.96 (0.36–2.57)	0.94	1.14 (0.35–3.69)	0.81
CD4+ count at respective ART initiation (cells/mm^3^)												
> 350	Ref		Ref		Ref		Ref		Ref		Ref	
200–349	2.74 (1.41–5.33)	0.003	2.18 (1.09–4.36)	0.02	1.76 (0.84–3.68)	0.12	1.80 (0.86–3.76)	0.11	3.98 (0.81–19.51)	0.08	4.22 (0.89–19.94)	0.07
< 200	4.90 (2.58–9.30)	< 0.001	3.75 (1.91–7.37)	< 0.001	3.04 (1.49–6.18)	0.002	3.03 (1.48–6.17)	0.002	8.80 (2.05–37-71)	0.003	9.95 (2.38–41.60)	0.002
Residence												
Semi-urban or rural	Ref		Ref		Ref		Ref		Ref		Ref	
Urban	1.56 (1.15–2.11)	0.004	1.53 (1.13–2.07)	0.006	1.38 (1.01–1.88)	0.03	1.42 (1.04–1.94)	0.02	4.52 (1.03–19.70)	0.04	4.60 (0.96–21.86)	0.05
ART												
First-line	Ref		Ref		–	–	–	–	–	–	–	–
Second-line	0.34 (0.21–0.56)	< 0.001	0.48 (0.28–0.82)	0.008	–	–	–	–	–	–	–	–
Duration on first-line ART prior to second-line ART initiation												
Per-year increase	–	–	–	–	–	–	–	–	1.00 (0.99–1.01)	0.10	1.00 (0.99–1.00)	0.88

*SHR* sub distribution hazard ratio, *CI* Confidence interval, *Ref* Reference group. All multivariable analyses included age, sex, CD4+ cell counts and residence. The overall multivariable analysis additionally included type of ART

A subset of 150 (17%) participants receiving second-line ART had data available on CD4+ cell counts at the time of their first-line ART initiation. The median (IQR) CD4+ count at first-line ART initiation among participants receiving second-line ART was 382 (218–571) cells/mm^3^. Higher CD4+ cell counts at first-line ART initiation were associated with lower risk of TB during second-line ART receipt (SHR = 0.97 per unit higher CD4+ cell count, 95%CI 0.95–0.99, *p* = 0.008). Kaplan-Meier survival curves for the proportion of these participants who remained free of TB after stratifying by CD4+ cell counts at initiation of first-line ART are depicted in Additional file 1: Fig. S1.

## Discussion

To our knowledge, our report is the largest programmatic study to estimate the incidence rate of TB among adult PLHIV receiving first- and second-line ART prior to widespread IPT implementation in India. We estimated an incidence rate of 4.39 TB cases per 100 person-years among participants on first-line ART, which was significantly higher than the estimated incidence rate of 1.64 TB cases per 100 person-years among participants on second-line ART. We additionally found an association between low CD4+ cell counts at ART initiation, male sex and urban residence, and higher risk of TB in participants on first- and second-line ART.

While the risk of TB on first-line ART has been reported in many settings [9], few studies have evaluated TB risk during second-line ART and none have estimated the incidence rate of TB during second-line ART in India [10]. Our study addresses this knowledge-gap by estimating age, sex and CD4+ cell count stratified incidence rates of TB in PLHIV receiving PI based second-line ART regimens under programmatic conditions in western India. We found a lower risk of TB among participants receiving second-line ART compared to those on first-line ART, independent of age, sex, residence, CD4+ cell counts and competing risks of LTFU and death. While our study results are consistent with prior reports of a switch to PI based regimens reducing the incidence of opportunistic infections and mortality in PLHIV with virologic failure [11–13], a survivor bias likely to be present in those who initiated second-line ART after failing prior regimens, or a delay in switching to second-line ART may have contributed to our study findings. Our estimated incidence rate of TB on first-line ART is comparable to that reported from studies in South Africa [14–16] and one study in western India [17], but is lower than the incidence rate previously reported from other Indian cohorts [18, 19]. Undiagnosed TB in participants on first-line ART who died in our study and, lower CD4+ cell counts at ART initiation, oversampling of male participants and the inclusion of prevalent TB in estimating disease burden in prior studies may explain some of these differences.

Immune suppression has long been associated with TB and prior studies in HIV-infected individuals have shown a dose-response relationship between CD4+ cell counts, particularly at ART initiation, and risk of TB [20–23]. While we did not evaluate time-updated CD4+ cell counts, we found a similar association between low CD4+ cell counts at ART initiation, particularly CD4+ under 200 cells/mm^3^, and higher risk of TB during follow-up. Further, we found a higher risk of TB during second-line ART in participants with lower CD4+ cell counts at their first-line ART initiation. Overall, 50% of all TB cases in our study occurred within 6 months of first- or second-line ART initiation and the incidence rate of TB was highest during the first 3 months of ART. Unmasking of subclinical TB among participants with low CD4+ cell counts may partly explain our study findings. Subclinical TB is difficult to diagnose and a recent clinical trial did not find a significant health benefit of empiric TB therapy over IPT in advanced HIV disease [24]. Consistent with prior recommendations, our results suggest systematic screening for TB and prioritization of IPT in HIV-infected adults with low CD4+ cell counts.

Male participants had higher risk of TB compared to their female counterparts during first-line ART. This may partly be attributed to males having more advanced immunosuppression at ART initiation compared to females. Our finding may also reflect the global epidemiology of TB where inherent biological or social differences may account for a higher burden of TB in males compared to females [25, 26]. Furthermore, we did not evaluate exposure to alcohol and tobacco smoke, important risk factors of TB over-represented in males, which may have accounted for a higher risk of TB compared to females. Finally, participants residing in an urban region had higher risk of TB compared to those with a semi-urban or rural residence. This finding may be indicative of the underlying transmission dynamics of TB due to overcrowding and social networks which may differ between urban and non-urban regions.

Our study has limitations. The median follow-up time in our cohort was 13 months and nearly 26% of participants were transferred to another ART center during follow-up. While these participants did not have TB at the time of transfer, those with a rural or semi-urban residence were more likely to be transferred leading to shorter observed follow-up time and possible underestimation of TB incidence in this group. Similarly, nearly 13% of participants were lost to follow-up which was more likely to occur in males and those with CD4+ counts less than 200 cells/mm^3^. While we partially addressed this limitation by accounting for loss to follow-up and death as competing risks, a differential outcome ascertainment may have underestimated the incidence rate of TB in our study. Furthermore, information on CD4+ cell counts at follow-up visits, VL at the time of TB diagnosis or second-line ART initiation, *M.tuberculosis* infection and contact with a known TB case, and smoking were unavailable, limiting the assessment of key risk factors for TB in our study population. Finally, prevalent and incident TB disease was diagnosed according to standard programmatic guidelines which rely on symptom screening followed by smear microscopy. Culture confirmation of TB disease was lacking and we may have underestimated the incidence of TB disease in our study.

## Conclusion

Our study leverages programmatic data from a large sample size of HIV-infected adults to estimate the incidence rate of TB during first- and second-line ART prior to widespread IPT. In doing so, we have provided an estimate of TB risk in a real-world setting to serve as a reference for evaluating the effectiveness of the ongoing IPT rollout in ART clinics in India. Additionally, we used competing risks regression to account for some of the limitations of retrospective analysis of cohort data where loss to follow-up and death may prevent the accurate ascertainment of TB during first- and second-line ART; thereby identifying high-risk individuals for IPT prioritization. Risk of tuberculosis was lower in PLHIV receiving second-line ART compared to first-line ART. Prioritizing IPT in PLHIV with low CD4+ counts, urban residence and in males may further mitigate the risk of tuberculosis during ART.

## Supplementary information

**Additional file 1: Fig. S1.** Proportion of participants on second-line ART surviving free of incident TB disease stratified by CD4+ cell counts at their first-line ART initiation. Cmm – cubic millimeter, TB – tuberculosis, ART – antiretroviral therapy.

## Abbreviations

AFB: Acid fast bacilli
ART: Antiretroviral therapy
BJGMC: Byramjee-Jeejeebhoy Government Medical College
CI: Confidence interval
IPT: Isoniazid preventive therapy
IQR: Interquartile range
IR: Incidence rate
LTFU: Loss to follow-up
NACO: National AIDS Control Organization
NNRTI: Non-nucleotide reverse transcriptase inhibitor
PI: Protease inhibitor
PLHIV: People living with HIV
SGH: Sassoon General Hospitals
SHR: Sub-distribution hazard ratios
TB: Tuberculosis
VL: Viral load
WHO: World Health Organization
