# Supplementary figures and images for: Incidence of tuberculosis in HIV-infected adults on first- and second-line antiretroviral therapy in India
Source: BMC Infect Dis. 2019 Oct 29;19:914. doi: 10.1186/s12879-019-4569-z (PMC6820927; doi:10.1186/s12879-019-4569-z)

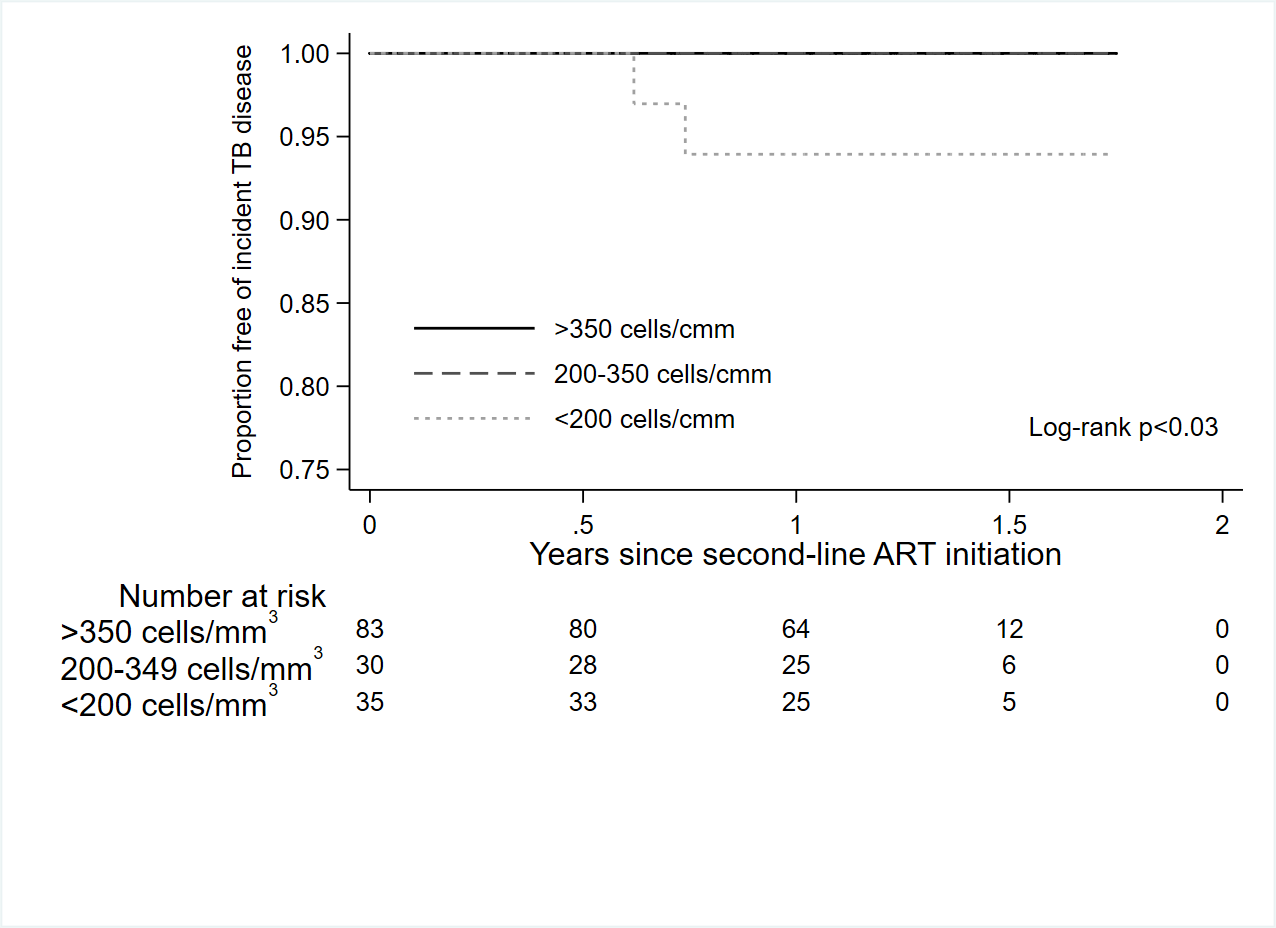

Supplement: Supplementary file 1 — Additional file 1: Fig. S1. Proportion of participants on second-line ART surviving free of incident TB disease stratified by CD4+ cell counts at their first-line ART initiation. Cmm – cubic millimeter, TB – tuberculosis, ART – antiretroviral therapy. [file 12879_2019_4569_MOESM1_ESM.tif]
